# Supplementary figures and images for: Social inequalities, length of hospital stay for chronic conditions and the mediating role of comorbidity and discharge destination: A multilevel analysis of hospital administrative data linked to the population census in Switzerland
Source: PLoS One. 2022 Aug 24;17(8):e0272265. doi: 10.1371/journal.pone.0272265 (PMC9401154; doi:10.1371/journal.pone.0272265)

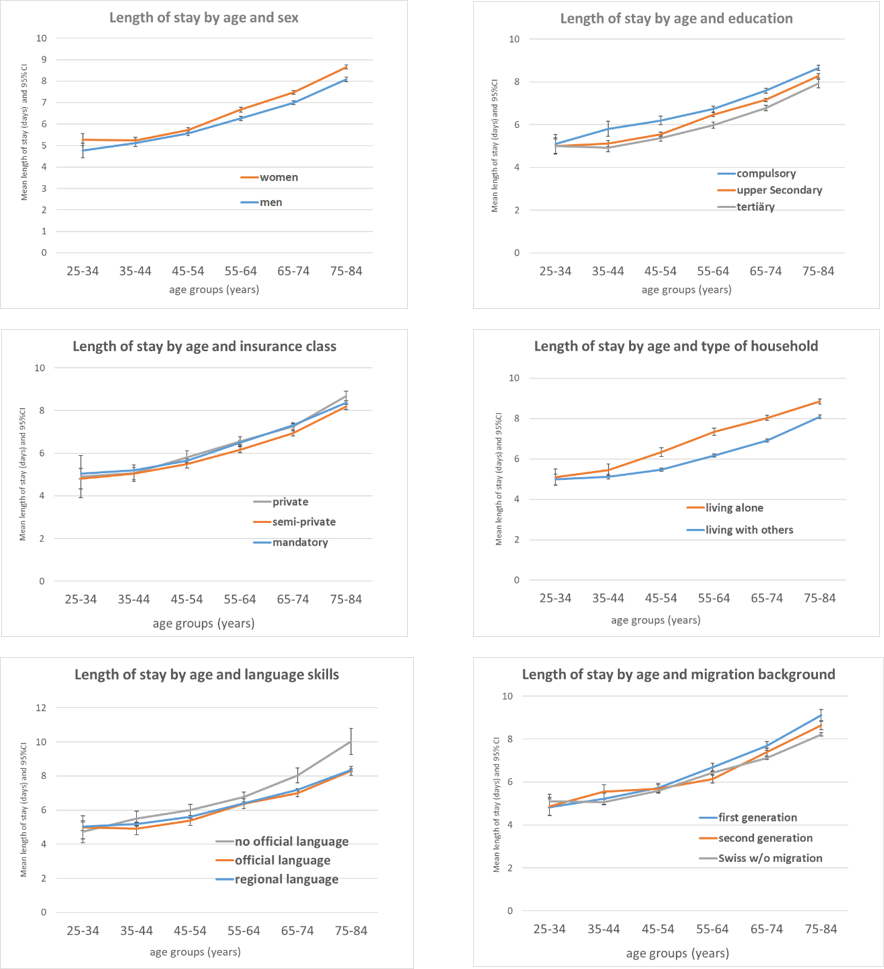

Supplement: S1 Fig — Length of stay by age and social factors (top left: sex; top right: education; middle left: insurance class, middle right: type of household; bottom right: language skills; bottom left: migration background. (TIF) [file pone.0272265.s001.tif]

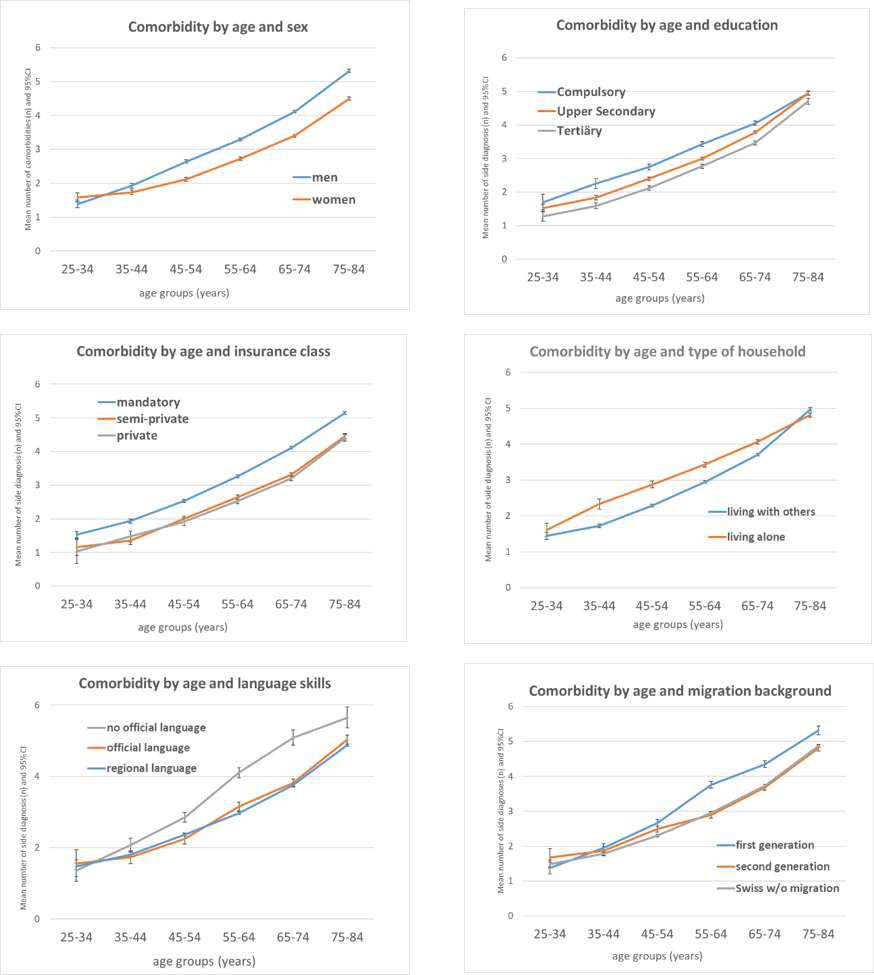

Supplement: S2 Fig — Comorbidity by age and social factors (top left: sex; top right: education; middle left: insurance class, middle right: type of household; bottom right: language skills; bottom left: migration background). (TIF) [file pone.0272265.s002.tif]

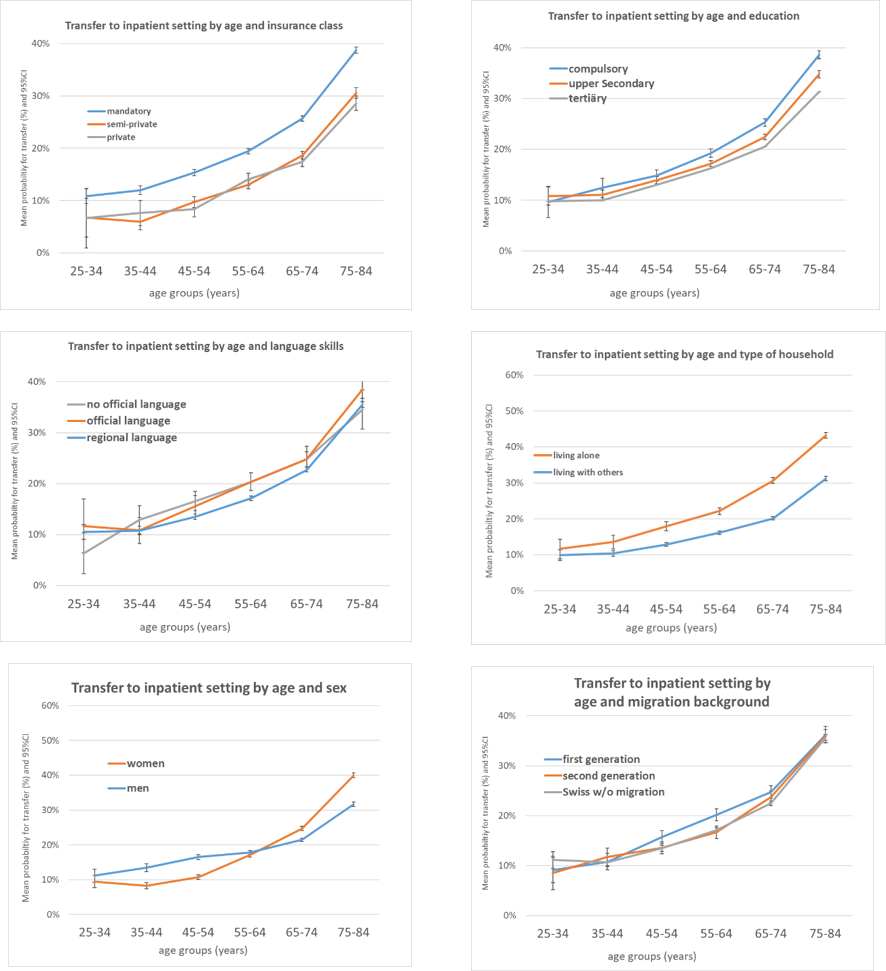

Supplement: S3 Fig — Discharge destination by age and social factors (top left: sex; top right: education; middle left: insurance class, middle right: type of household; bottom right: language skills; bottom left: migration background). (TIF) [file pone.0272265.s003.tif]
