# Supplementary material for: Social inequalities, length of hospital stay for chronic conditions and the mediating role of comorbidity and discharge destination: A multilevel analysis of hospital administrative data linked to the population census in Switzerland
Source: PLoS One. 2022 Aug 24;17(8):e0272265. doi: 10.1371/journal.pone.0272265 (PMC9401154; doi:10.1371/journal.pone.0272265)
Supplement: S1 Table — (PDF) [file pone.0272265.s004.pdf]

**S1 Table. Cluster sizes and Intra-Class-Correlation (ICC) of cluster Variables, linear CCMM (null-model with outcome length of stay).**

| Custer Variable | Records (N) | Clusters (N) | Cluster Size | | | | | ICC |
|---|---|---|---|---|---|---|---|---|
| | | | Min. | Max. | Median | Mean | SD | |
| Hospitals | 141’307 | 188 | 1 | 5’513 | 321.50 | 751.63 | 1’085.96 | 0.281 |
| Patients | 141’307 | 92’623 | 1 | 23 | 1.00 | 1.53 | 1.02 | 0.154 |
