# Supplementary material for: Social inequalities, length of hospital stay for chronic conditions and the mediating role of comorbidity and discharge destination: A multilevel analysis of hospital administrative data linked to the population census in Switzerland
Source: PLoS One. 2022 Aug 24;17(8):e0272265. doi: 10.1371/journal.pone.0272265 (PMC9401154; doi:10.1371/journal.pone.0272265)
Supplement: S2 Table — The least significant difference adjusted significance level is 0.05. 1) cf. Table 4; 2) cf. Table 5. (PDF) [file pone.0272265.s005.pdf]

S2 Table. Effect estimates for significant interactions between main diagnosis and social factors.

Simple Contrast for significant interactions between main diagnosis and social factors		Contrast Estimate	Adj. Sig.	95% Confidence Interval	
**Main Diagnosis**	**Educational Attainment**			Lower	Upper
Other main diagnoses[1)]	Mandatory vs Tertiary	0.05	0.29	-0.04	0.14
	Upper Secondary vs Tertiary	0.04	0.38	-0.04	0.12
Colon Cancer	Mandatory vs Tertiary	0.99	0.02	0.13	1.84
COPD	Upper Secondary vs Tertiary	0.56	0.04	0.02	1.10
Asthma	Mandatory vs Tertiary	0.93	0.04	0.06	1.80
Ischaemic HD	Upper Secondary vs Tertiary	-0.17	0.04	-0.33	-0.01
**Main Diagnosis**	**Hospital insurance class**				
Other main diagnoses[1)]	private vs. general	0.36	<0.001	0.18	0.53
	semi-private vs general	0.15	0.03	0.02	0.29
Colon Cancer	semi-private vs general	-1.10	0.00	-1.85	-0.35
Breast Cancer	private vs. general	-0.56	0.01	-0.99	-0.13
	semi-private vs general	-0.36	0.03	-0.67	-0.04
AMI	private vs. general	0.65	0.01	0.20	1.10
	semi-private vs general	0.54	0.00	0.20	0.87
COPD	private vs. general	1.50	0.00	0.49	2.50
	semi-private vs general	0.92	0.01	0.27	1.58
Back problems	private vs. general	0.95	0.00	0.46	1.44
	semi-private vs general	0.61	0.00	0.28	0.93
**Main diagnosis**	**Type of household**				
Other main diagnoses[1)]	living alone vs living with others	0.28	<0.001	0.20	0.37
Lung cancer	living alone vs living with others	0.92	0.000	0.46	1.38
Colon Cancer	living alone vs living with others	1.21	0.001	0.49	1.94
Back problems	living alone vs living with others	0.56	0.000	0.30	0.82
**Main diagnosis**	**Languag skills**				
Other main diagnoses[2)]	allophone vs. regional language	0.34	<0.001	0.13	0.55
	no regional vs. regional language	0.00	0.98	-0.12	0.13
Colon cancer	allophone vs. regional language	-1.559	0.06	-3.16	0.04
COPD	allophone vs. regional language	-0.749	0.06	-1.54	0.04
Ischaemic HD	no regional vs. regional language	0.345	0.01	0.10	0.59
**Main diagnosis**	**Migration background**				
Other main diagnoses[2)]	1st Generation vs Swiss w/o migr.background	0.16	<0.001	0.05	0.27
	2nd Generation vs Swiss w/o mig. background	0.02	0.68	-0.07	0.10
Colon Cancer	2nd Generation vs Swiss w/o mig. background	-1.28	0.005	-2.17	-0.40
Ostheoarthritis	1st Generation vs Swiss w/o migr.background	0.02	0.838	-0.16	0.20
Back problems	2nd Generation vs Swiss w/o mig. background	0.39	0.011	0.09	0.69

The least significant difference adjusted significance level is .05.

[1)] cf tabel 4; [2)] cf tabel 5
