# Supplementary material for: Social inequalities, length of hospital stay for chronic conditions and the mediating role of comorbidity and discharge destination: A multilevel analysis of hospital administrative data linked to the population census in Switzerland
Source: PLoS One. 2022 Aug 24;17(8):e0272265. doi: 10.1371/journal.pone.0272265 (PMC9401154; doi:10.1371/journal.pone.0272265)
Supplement: S3 Table — β: Difference in average number of comorbidities to the respective reference category estimated from the mixed linear regression model containing all variables listed in the table and random effects for hospitals and patients. +centred by chronic conditions; *Controlling for clustering on hospital- and patient-level and adjusted for age, chronic condition, language region of hospital and year of discharge. (PDF) [file pone.0272265.s006.pdf]

S3 Table. Associations of comorbidity with social factors (Linear CCMM).

**Outcome:** N° of side diagnosis+	**Modell Comorbidity** (N=140'903)			
**Fixed Effects***	β (N)	p-value	95% CI Lower	95% CI Upper
Intercept	2.70	0.109	-0.60	6.00
Educational attainement				
Compulsory	0.37	<0.001	0.33	0.41
Upper secondary	0.23	<0.001	0.19	0.26
Tertiary	Ref.			
Insurance Class				
Private	0.01	0.863	-0.06	0.07
Semi-private	-0.05	0.115	-0.11	0.01
Mandatory	Ref.			
Household type				
Living alone	0.22	<0.001	0.17	0.26
Living with others	Ref.			
Sex				
Men	0.23	<0.001	0.18	0.27
Women	Ref.			
Nationality				
Other nationality	0.32	<0.001	0.21	0.42
EU/EFTA	0.02	0.507	-0.04	0.08
Swiss	Ref.			

β: Difference in average number of comorbidities to the respective reference category estimated from the mixed linear regression model containing all variables listed in the table and random effects for hospitals and patients.

+ centred by chronic conditions

*Controlling for clustering on hospital- and patient-level and adjusted for age, chronic condition, language region of hospital and year of discharge"
