# Supplementary material for: Social inequalities, length of hospital stay for chronic conditions and the mediating role of comorbidity and discharge destination: A multilevel analysis of hospital administrative data linked to the population census in Switzerland
Source: PLoS One. 2022 Aug 24;17(8):e0272265. doi: 10.1371/journal.pone.0272265 (PMC9401154; doi:10.1371/journal.pone.0272265)
Supplement: S4 Table — *Controlling for clustering on hospital- and patient-level and adjusted for age, chronic condition, language region of hospital and year of discharge. (PDF) [file pone.0272265.s007.pdf]

S4 Table. Associations of discharge destination with social factors and factors related to hospital stay (Logistic CCMM).

**Binary Outcome:** Transfer to inpatient setting (vs. discharge to home)	**Model Discharge Destination (N=140'903)**						
	β	95% CI		OR	p-value	95% CI	
**Fixed Effects***		Lower	Upper			Lower	Upper
Intercept	-1.86	-6.66	2.95	0.16	0.449	0.00	19.11
Educational attainement							
Compulsory	0.01	-0.05	0.07	1.01	0.708	0.95	1.07
Upper secondary	0.00	-0.05	0.05	1.00	0.973	0.95	1.05
Tertiary	Ref.						
Insurance Class							
Private	-0.08	-0.21	0.04	0.92	0.196	0.81	1.04
Semi-private	-0.10	-0.20	-0.01	0.90	0.030	0.82	0.99
Mandatory	Ref.						
Household type							
Living alone	0.45	0.40	0.50	1.56	<0.001	1.49	1.64
Living with others	Ref.						
Sex							
Men	-0.29	-0.33	-0.25	0.75	<0.001	0.72	0.78
Women	Ref.						
Nationality							
Other nationality	0.09	-0.02	0.20	1.09	0.116	0.98	1.22
EU/EFTA	0.05	0.00	0.10	1.05	0.046	1.00	1.10
Swiss	Ref.						
Comorbidity							
NSD (centered by CC)	0.11	0.10	0.13	1.12	<0.001	1.10	1.14
Psychic comorbidity: yes	0.34	0.28	0.40	1.40	<0.001	1.33	1.49
Psychic comorbidity: no	Ref.						
Hospital Ward							
Surgical	0.29	0.09	0.49	1.34	0.004	1.10	1.64
Internal medicine or other	Ref.						
Need of intensive care							
Yes	0.68	0.47	0.90	1.98	<0.001	1.60	2.45
No	Ref.						

*Controlling for clustering on hospital- and patient-level and adjusted for age, chronic condition, language region of hospital and year of discharge
