# Supplementary material for: Social inequalities, length of hospital stay for chronic conditions and the mediating role of comorbidity and discharge destination: A multilevel analysis of hospital administrative data linked to the population census in Switzerland
Source: PLoS One. 2022 Aug 24;17(8):e0272265. doi: 10.1371/journal.pone.0272265 (PMC9401154; doi:10.1371/journal.pone.0272265)
Supplement: S5 Table — The regression coefficients β are the estimated differences in average length of stay between the respective category and the reference category obtained from the respective model. *The model controls for clustering on hospital- and on patient-level and is adjusted for language region of hospital and year of discharge. (PDF) [file pone.0272265.s008.pdf]

S5 Table. Association of length of stay with demographic factors (age implemented as categorical variable), social factors, health status and factors related to hospital stay (linear CCMM D).

Outcome: LOS (days)	Model D - age categorical (N=140'903)			
	β (days)	p-value	95% CI	
**Fixed Effects***			Lower	Upper
Intercept	2.25	<0.001	1.34	3.15
Educational attainement				
Compulsory	0.05	0.25	-0.04	0.14
Upper secondary	0.04	0.35	-0.04	0.12
Tertiary	Ref.			
Insurance Class				
Private	0.36	<0.001	0.18	0.54
Semi-private	0.16	0.02	0.02	0.29
Mandatory	Ref.			
Household type				
Living alone	0.28	<0.001	0.20	0.37
Living with others	Ref.			
Sex				
Men	-0.30	<0.001	-0.38	-0.22
Women	Ref.			
Age				
75-84	0.67	<0.001	0.31	1.03
65-74	0.55	<0.001	0.23	0.86
55-64	0.47	<0.001	0.23	0.71
45-54	0.27	<0.001	0.09	0.46
25-44	Ref.			
Nationality				
Other nationality	0.21	0.04	0.01	0.41
EU/EFTA	0.14	0.02	0.02	0.26
Swiss	Ref.			
Chronic Condition (CC)				
Lung cancer	4.81	<0.001	4.38	5.24
Colon cancer	6.97	<0.001	6.27	7.67
Breast cancer	1.53	<0.001	1.17	1.89
Prostata cancer	2.54	<0.001	1.97	3.11
Diabetes w/o complication	4.03	<0.001	3.04	5.02
Diabetes with complication	7.49	<0.001	6.79	8.19
Acute myocardial infarction	0.70	<0.001	0.39	1.02
Acute cerebrovascular diseases	3.92	<0.001	3.41	4.43
Congestive heart failure	6.41	<0.001	5.93	6.90
COPD	5.09	<0.001	4.61	5.57
Asthma	2.00	<0.001	1.63	2.37
Ostheoarthritis	2.93	<0.001	2.38	3.49
Back problems	3.50	<0.001	3.00	3.99
Disc disorder	2.51	<0.001	1.93	3.09
Ischämic heart disease	Ref.			

Comorbidity				
NSD (centered by CC)	0.80	<0.001	0.70	0.89
Psychic comorbidity: yes	0.36	<0.001	0.14	0.57
Psychic comorbidity: no	Ref.			
Hospital Ward				
Surgical	1.72	<0.001	1.24	2.21
Internal medicine or other	Ref.			
Need of intensive care				
Yes	3.15	<0.001	2.78	3.53
No	Ref.			
Discharge destination				
Died in hospital	0.34	0.31	-0.32	1.01
Transfer to inpatient setting	1.99	0.00	1.76	2.22
Discharge to home	Ref.			
Akaike criterion, corrected	868'829			

The regression coefficients β are the estimated differences in average length of stay between the respective category and the reference category obtained from the respective model.
*The model controls for clustering on hospital- and on patient-level and is adjusted for language region of hospital and year of discharge"
