# Supplementary material for: Social inequalities, length of hospital stay for chronic conditions and the mediating role of comorbidity and discharge destination: A multilevel analysis of hospital administrative data linked to the population census in Switzerland
Source: PLoS One. 2022 Aug 24;17(8):e0272265. doi: 10.1371/journal.pone.0272265 (PMC9401154; doi:10.1371/journal.pone.0272265)
Supplement: S1 File — (PDF) [file pone.0272265.s009.pdf]

S1_File

Auszug Variablen-Liste, BFS-Daten

Variables of the SIHOS-Database used for the paper *«Social inequalities, length of hospital stay for chronic conditions and the mediating role of comorbidity and discharge destination: A multilevel analysis of hospital administrative data linked to the population census in Switzerland»*, *Bayer-Oglesby et al. 2022*

Benötigte Daten

BFS-Daten (falls im Antrag vorgesehen)

1. *Benötigte Variablen (diese sind vollständig aufzulisten, wobei sich die Schreibweise nach dem entsprechenden Datenkatalog richten muss)*
2. *Referenzperiode*
3. *Verknüpfungsidentifikator*

Es müssen Variablen aus den folgenden Datenquellen verknüpft werden:
- STATPOP
- Strukturerhebung
- Medizinische Statistik der Krankenhäuser (MS)
- MS: Verknüpfung über die Jahre (Mehrfachaufenthalte)

1) Benötigte Variablen

**STATPOP-Nr**	**Bezeichnung**	**Kategorien/Bemerkung**
	anonymiserter individueller Identifikator	
Abgeleitet aus 31	Alter	in Jahren
33	Geschlecht	1 weiblich<br>2 männlich
412	Staatsangehörigkeit	1 Schweiz<br>2 EU/EFTA<br>3 Anderer europäischer Staat<br>4 Aussereuropäischer Staat<br>5 Unbekannt
624	Haushaltsart	1 Privathaushalt
**Struktur-erhebung-Nr.**	**Bezeichnung**	**Kategorien/Bemerkung**
	anonymisierter individueller Identifikator	
1	1. Hauptsprache (sprache_1)<br>2. Hauptsprache (sprache_2)<br>3. Hauptsprache (sprache_3)	1 Deutsch<br>2 Französisch<br>3 Italienisch<br>4 Englisch<br>5 Andere Sprachen
6 und 7	Migrationshintergrund	1 Schweizer/-in ohne Migrationshintergrund<br>2 Schweizer-/in mit Migrationshintergrund<br>3 Ausländer/-in der ersten Generation<br>4 Ausländer/-in der zweiten und höheren Generation<br>5 Migrationshintergrund unbekannt
8	höchste abgeschlossene Ausbildung	1 Sekundarstufe 1<br>2 Sekundarstufe 2<br>3 Tertiärstufe
Abgeleitet	Haushaltstyp	1 Einpersonenhaushalte<br>2 Nichtfamilienhaushalte mit mehreren Personen<br>3 Paare ohne Kinder im Haushalt<br>4 Paare mit Kindern im Haushalt<br>5 Elternteile mit Kindern im Haushalt<br>6 Mehrfamilienhaushalte
**MS Variablen Nr.**	**Bezeichnung**	**Bemerkung**
**Einschlusskriterien:**<br>Teilnahme des Patienten/der Patientin an einer der Strukturerhebungen 2010-2014 UND 1.3.V01 (Behandlungsart) = 3 (stationär) UND (Statistikfall = A oder Statistikfall=B) , mit allen Wiedereintritten		
**Minimaldatensatz (MB-Datensatz)**		

0.2.V01a	anonymisierter individueller Identif-ikator	
ID	Anonyme Fallnummer	
0.0.V01	Jahr	Um zeitliche Trends in den Datenanalysen zu berücksichtigen
EintrittEr-hebungs-jahr	Eintritt erfolgte im Erhebungsjahr MS	Für analysenspezifische Selektion der Fälle
Austritt Er-he-bungsjahr	Austritt erfolgte im Erhebungsjahr MS	Für analysenspezifische Selektion der Fälle
**0.1.V02a**	Anonyme Bertriebsnummer	Damit berücksichtigt werden kann, dass Hospi-talisationen im gleichen Spital einen Cluster bilden (z.B. in Random-Effects-Modellen)
**0.2.V02**	Kennzeichnung des Statistikfalls	A, B, C; für Selektion Fälle
0.3.V03	Patientengruppen-Datensatz	
1.1.V01	Geschlecht	Für Plausibilisierung mit STATPOP
1.1.V03	Alter bei Eintritt in Jahren	Für Plausibilisierung mit STATPOP
0.0.V12	Schweizer - Nicht-Schweizer	Für Plausibilisierung mit STATPOP
1.3.V01	Behandlungsart	Für Selektion Fälle
1.3.V02	Klasse	
1.3.V03	Aufenthalt in Intensivstation	
1.4.V01	Hauptkostenstelle	
1.5.V03	Aufenthalt nach Austritt	
0.0.V05-2	Aufenthaltsdauer (SwissDRG)	
**Patientengruppen-Zusatzdaten (MD-Datensatz)**		
	ID Anonyme Fallnummer	
4.2.V010	MD-Hauptdiagnose	ICD-10-GM-Kode
4.2.V020	MD-Zusatz zu Hauptdiagnose	ICD-10-GM-Kode
4.2.V030<br>4.2.V040<br>4.2.V…	MD-1.Nebendiagnose<br>MD-2. Nebendiagnose<br>etc. für alle Nebendiagnosen	ICD-10-GM-Kode
**Sequenzvariablen**		
InputFile	Herkunftsfile des Records	0=MS;
s_ms	Sequenznummer der MS-Fälle (abgeleitet von 1.2.V01/ 1.5.V0)	Nr. der Behandlungssequenz unter Berück-sichtigung der MD-Kennzeichnung von Wie-dereintritten (Zwischenaustritte 4.7.V01, 4.7.V11 etc. und Wiedereintritte 4.7.V.02, 4.7.V12 etc.), inkl. Grund der Wiedereintritte (4.7.V03, 4.7.V13 etc.)

2. Referenzperioden:

- STATPOP/Strukturerhebung: 2010 – 2014
- Medizinische Statistik: 2010 − 2016

3. Verknüpfungsidentifikator für die Verknüpfung

- von Strukturerhebung und STATPOP: AHVN13
- von Strukturerhebung / STATPOP mit MS: anonymer Verbildungscode, der für die STATPOP und Strukturerhebung via Stichprobenregister gebildet werden kann.

Bedingungen:

Die Daten werden ausschliesslich für den im Vertrag definierten Zweck verwendet und nicht an Dritte wei-tergegeben. Die Daten werden nach Abschluss des Projekts gelöscht.

Die Daten werden an einem gesicherten Ort gespeichert, der ausschliesslich den Projektmitarbeitenden Zugang erlaubt.
