# Supplementary material for: Social inequalities, length of hospital stay for chronic conditions and the mediating role of comorbidity and discharge destination: A multilevel analysis of hospital administrative data linked to the population census in Switzerland
Source: PLoS One. 2022 Aug 24;17(8):e0272265. doi: 10.1371/journal.pone.0272265 (PMC9401154; doi:10.1371/journal.pone.0272265)
Supplement: Attachment — Submitted filename: Response to Reviewers.docx [file pone.0272265.s011.docx]

PONE-D-21-31664

Social inequalities, length of hospital stay for chronic conditions and the mediating role of comorbidity and discharge destination: A multilevel analysis of hospital administrative data linked to the population census in Switzerland

Journal Requirements:

Please review your reference list to ensure that it is complete and correct. If you have cited papers that have been retracted, please include the rationale for doing so in the manuscript text, or remove these references and replace them with relevant current references. Any changes to the reference list should be mentioned in the rebuttal letter that accompanies your revised manuscript. If you need to cite a retracted article, indicate the article’s retracted status in the References list and also include a citation and full reference for the retraction notice.

***Changes to the reference list***

*Added references*

*47.	Bachmann N, Zumbrunn A, Bayer-Oglesby L. Social and Regional Factors Predict the Likelihood of Admission to a Nursing Home After Acute Hospital Stay in Older People With Chronic Health Conditions: A Multilevel Analysis Using Routinely Collected Hospital and Census Data in Switzerland. Frontiers in Public Health. 2022;10:871778*

*48.	Zumbrunn A, Bachmann N, Bayer-Oglesby L, Joerg R. Social disparities in unplanned 30-day readmission rates after hospital discharge in patients with chronic health conditions: A retrospective cohort study using patient level hospital administrative data linked to the population census in Switzerland [Internet]. medRxiv; 2022 [cited 2022 Jan 25]. Available from:* [*https://www.medrxiv.org/content/10.1101/2022.01.18.22269480v1*](https://www.medrxiv.org/content/10.1101/2022.01.18.22269480v1)

*71.	Lee SW. Regression analysis for continuous independent variables in medical research: statistical standard and guideline of Life Cycle Committee. Life Cycle [Internet]. 2022 [cited 2022 Jun 20];2. Available from:* [*http://www.elifecycle.org/archive/view_article?pid=lc-2-0-3*](http://www.elifecycle.org/archive/view_article?pid=lc-2-0-3)

*Corrections (as necessary):*

*All Journal name abbreviations are now those found in the* [*National Center for Biotechnology Information (NCBI) databases*](http://www.ncbi.nlm.nih.gov/nlmcatalog/journals)

*All published articles are now cited with traditional volume and page numbers*

Additional Editor Comments (if provided):

The Reviewers and I noted several issues not least of which was methodological concerns. I think that statistical reference may be needed (i.e., <https://doi.org/10.54724/lc.2022.e3>). Thank you.

***Response****: We have added the suggested reference at line 312. At line 164 we refer to the paper of Barker et al (2020), which includes recommendations for best practice of cross-classified multilevel analysis. In addition, we now cite Barker also at line 309. At lines 344 and 350 we refer to Hayes and Todd (2018) “Introduction to Mediation, Moderation, and Conditional Process Analysis”*

[Note: HTML markup is below. Please do not edit.]

Reviewers' comments:

Reviewer's Responses to Questions

**Comments to the Author**

1. If the authors have adequately addressed your comments raised in a previous round of review and you feel that this manuscript is now acceptable for publication, you may indicate that here to bypass the “Comments to the Author” section, enter your conflict of interest statement in the “Confidential to Editor” section, and submit your "Accept" recommendation.

Reviewer #3: All comments have been addressed

2. Is the manuscript technically sound, and do the data support the conclusions?

The manuscript must describe a technically sound piece of scientific research with data that supports the conclusions. Experiments must have been conducted rigorously, with appropriate controls, replication, and sample sizes. The conclusions must be drawn appropriately based on the data presented.

Reviewer #3: Partly

3. Has the statistical analysis been performed appropriately and rigorously?

Reviewer #3: No

4. Have the authors made all data underlying the findings in their manuscript fully available?

The [PLOS Data policy](https://eur03.safelinks.protection.outlook.com/?url=http%3A%2F%2Fwww.plosone.org%2Fstatic%2Fpolicies.action%23sharing&data=05%7C01%7Clucy.bayer%40fhnw.ch%7Cb5a9f6f25f154ed1b72108da4cea63e9%7C9d1a5fc8321e4101ae63530730711ac2%7C0%7C0%7C637906866833556711%7CUnknown%7CTWFpbGZsb3d8eyJWIjoiMC4wLjAwMDAiLCJQIjoiV2luMzIiLCJBTiI6Ik1haWwiLCJXVCI6Mn0%3D%7C3000%7C%7C%7C&sdata=dmQNQy43RMc05%2BxOsK9ZmxZDKD%2B7nhrU7%2FhXGk%2FPTak%3D&reserved=0) requires authors to make all data underlying the findings described in their manuscript fully available without restriction, with rare exception (please refer to the Data Availability Statement in the manuscript PDF file). The data should be provided as part of the manuscript or its supporting information, or deposited to a public repository. For example, in addition to summary statistics, the data points behind means, medians and variance measures should be available. If there are restrictions on publicly sharing data—e.g. participant privacy or use of data from a third party—those must be specified.

Reviewer #3: No

***Response****: We had updated the Data Availability Statement and it has passed the technical check (c.f. below)*

5. Is the manuscript presented in an intelligible fashion and written in standard English?

PLOS ONE does not copyedit accepted manuscripts, so the language in submitted articles must be clear, correct, and unambiguous. Any typographical or grammatical errors should be corrected at revision, so please note any specific errors here.

Reviewer #3: No

6. Review Comments to the Author

Please use the space provided to explain your answers to the questions above. You may also include additional comments for the author, including concerns about dual publication, research ethics, or publication ethics. (Please upload your review as an attachment if it exceeds 20,000 characters)

Reviewer #3: Comments were partially addressed but the document preparation is not following PLoS one criteria. figures and tables were not correctly cited and not well prepared.

***Response****: The manuscript has passed the basic formatting requirements of PLOS ONE, as confirmed by Oriel Jerome Delas Alas Vida, PLOS ONE on May 17, 2022 (see below). To our knowledge, this implies that figures and tables are cited according to PLOS formatting guidelines, that the tables are formatted according to PLOS formatting guidelines, that the figures have passed the Preflight Analysis and Conversion Engine (PACE) digital diagnostic tool,* [*https://pacev2.apexcovantage.com/*](https://pacev2.apexcovantage.com/) *and that the data availability statement has been checked.*

it needs revision and grammar errors are serious problems.

***Response****: We would appreciate quotation of the alleged grammar errors. The manuscript has undergone thorough proofreading by a native speaker who is teaching English at university level (*[*https://www.fhnw.ch/en/people/margaret-oertig*](https://www.fhnw.ch/en/people/margaret-oertig)*). The first author is bilingual and has published in international peer review journals such as American Journal of Epidemiology, Environmental Health Perspectives etc.*

the flow of ideas, neatness, result reporting form, and discussion are not as such good. statistical analysis is not clearly stated and does not answer the objective.
generally, the way that the manuscript is prepared is difficult to understand.

***Response****: We would have appreciated specific comments on why the reviewer #3* *considers the flow of ideas, neatness, and result reporting form “as such not to be good”. We do clearly state statistical analysis, following up-to-date recommendations (lines 303 ff.). We apply appropriate statistical methods to answer the research questions, stated at lines 135-146. We would have appreciated a detailed comment on why the reviewer #3 has difficulties to understand the manuscript and on how we could improve the paper. To clarify the context of the current analysis we have extended the paragraph on the SIHOS study and added two recent publications that made use of the SIHOS-database (lines 125-127):*

*“The SIHOS study investigated social disparities that may manifest at different stages of a hospitalization: before hospital admission [3], during the hospital stay (this analysis), at discharge [47] or after the hospital stay [48].”*

7. PLOS authors have the option to publish the peer review history of their article ([what does this mean?](https://eur03.safelinks.protection.outlook.com/?url=https%3A%2F%2Fjournals.plos.org%2Fplosone%2Fs%2Feditorial-and-peer-review-process%23loc-peer-review-history&data=05%7C01%7Clucy.bayer%40fhnw.ch%7Cb5a9f6f25f154ed1b72108da4cea63e9%7C9d1a5fc8321e4101ae63530730711ac2%7C0%7C0%7C637906866833556711%7CUnknown%7CTWFpbGZsb3d8eyJWIjoiMC4wLjAwMDAiLCJQIjoiV2luMzIiLCJBTiI6Ik1haWwiLCJXVCI6Mn0%3D%7C3000%7C%7C%7C&sdata=wvt2b77y6xkUDBY70PsTDrkGFhSZW47brPPnXM9hvhA%3D&reserved=0)). If published, this will include your full peer review and any attached files.


If you choose “no”, your identity will remain anonymous, but your review may still be made public.


**Do you want your identity to be public for this peer review?** For information about this choice, including consent withdrawal, please see our [Privacy Policy](https://eur03.safelinks.protection.outlook.com/?url=https%3A%2F%2Fwww.plos.org%2Fprivacy-policy&data=05%7C01%7Clucy.bayer%40fhnw.ch%7Cb5a9f6f25f154ed1b72108da4cea63e9%7C9d1a5fc8321e4101ae63530730711ac2%7C0%7C0%7C637906866833556711%7CUnknown%7CTWFpbGZsb3d8eyJWIjoiMC4wLjAwMDAiLCJQIjoiV2luMzIiLCJBTiI6Ik1haWwiLCJXVCI6Mn0%3D%7C3000%7C%7C%7C&sdata=Ji9ncD%2BYQMmjGQ2yG3mObbzFmT8ykUgPqcCcN3dH31U%3D&reserved=0).

Reviewer #3: No
